# Between the invisible and the measurable: what GBD 2023 data reveal (and conceal) about neurodevelopmental disorders in South America

**DOI:** 10.1055/s-0046-1823679

**Published:** 2026-07-21

**Authors:** Erasmo Barbante Casella

**Affiliations:** 1 Universidade de São Paulo, Faculdade de Medicina, Hospital das Clínicas, Instituto da Criança, Departamento de Pediatria, São Paulo SP, Brazil.

Neurodevelopmental disorders (NDDs) have occupied a paradoxical position in public health agendas for decades: they are highly prevalent, exert a lasting impact across the life course, and yet remain partially invisible within surveillance systems especially in regions marked by structural inequalities. The present study, by analyzing trends from 1990 to 2023 based on estimates from the Global Burden of Disease (GBD 2023), provides a rare longitudinal and comparative view across 12 South American countries, while simultaneously highlighting the limits of what we are able to observe. 1

Three central findings consistently emerge:

- a modest increase in the agestandardized prevalence of autism spectrum disorder (ASD);
- relative stability in attention-deficit/hyperactivity disorder (ADHD); and
- a sustained decline in idiopathic developmental intellectual disability (IDID).

At first glance, these patterns may suggest distinct and even independent epidemiological trajectories. However, a more critical reading indicates that these trends are largely shaped not only by real changes in incidence or risk factors, but primarily by the capacity of health systems to detect, classify, and record these conditions.

The increase in ASD, although consistent, is modest when compared to highincome countries. This should not be interpreted as lower actual growth, but rather as a reflection of persistent underdiagnosis. The expansion of diagnostic criteria and increased global awareness appear to have a diminished impact in contexts where there is a shortage of specialists, standardized instruments, and well-structured care pathways. Thus, the observed increase may represent only the “tip of the iceberg,” revealing more about gradual advances in recognition than about true changes in the occurrence of the disorder.

In contrast, the stability of ADHD raises an important question: does it reflect true epidemiological stability, or a balance between opposing forces? On one hand, greater recognition and broader diagnostic criteria; on the other, persistent underidentification especially among vulnerable populations and outside formal school settings. Methodological heterogeneity between countries, and even within them, may mask meaningful variations, resulting in an apparent temporal neutrality.

The decline in IDID, in turn, is the most robust and biologically plausible finding from both a clinical and public health perspective. Improvements in perinatal conditions, access to prenatal care, childbirth assistance, and neonatal care represent interventions with direct impact on known risk factors for intellectual disability. This pattern reinforces the effectiveness of maternal and child health policies in the region. However, it also raises a complementary hypothesis: as preventable causes decrease, the relative proportion of cases with genetic or multifactorial etiologies may become more apparent, requiring new diagnostic and therapeutic paradigms.

One of the most revealing aspects of the study lies in sex differences. The persistence and, in the case of ASD, the possible widening of the male-to-female ratio suggests that we are not dealing solely with biological differences, but also with diagnostic biases. Girls and women remain underrepresented, possibly due to subtler clinical presentations, compensatory strategies, and lower referral rates for specialized evaluation. This finding underscores the urgent need to revise clinical models and screening instruments, historically calibrated for male profiles.

Beyond the numbers, the study exposes a critical issue: reliance on modeled estimates in contexts with scarce primary data. Although the GBD represents the gold standard in global comparability, its results are only as robust as the evidence that feeds them. In South America, epidemiological gaps persist, particularly in rural areas and lowincome populations. This implies that part of the observed trends may reflect model assumptions more than lived reality.

Another relevant dimension concerns environmental and social determinants. Exposure to pollutants, socioeconomic inequalities, and unequal access to education and healthcare all these factors interact in complex ways with biological vulnerabilities, shaping both risk and expression of NDDs. However, these dimensions remain insufficiently integrated into traditional epidemiological analyses, representing an important frontier for future research.

Given this scenario, several implications are unavoidable. First, it is necessary to strengthen developmental health surveillance by incorporating standardized instruments and early screening strategies into primary care. Second, professional training must be expanded and decentralized, reducing the concentration of expertise in major urban centers. Third, public policies must explicitly address barriers to access, including cost, distance, and fragmentation of care. Finally, it is imperative to invest in the local production of population-based epidemiological data capable of validating or challenging global estimates.

In summary, this study does more than describe trends, it reveals a mismatch: while we have advanced in the prevention of some conditions, we still fall short in identifying and caring for others. The challenge for the next decade will not be only to measure better, but to see better especially those who, until now, have remained at the margins of statistics.
